# Corrections

**DOI:** 10.1016/S1473-3099(15)00505-8

**Published:** 2016-01

*Tapia MD, Sow SO, Lyke KE, et al. Use of ChAd3-EBO-Z Ebola virus vaccine in Malian and US adults, and boosting of Malian adults with MVA-BN-Filo: a phase 1, single-blind, randomised trial, a phase 1b, open-label and double-blind, dose-escalation trial, and a nested, randomised, double-blind, placebo-controlled trial.* Lancet Infect Dis *2016; **16**: 31–42*—In the lower panel of figure 2A, the y axis read 0, 1, 10, 100, 100, 1000, and 10 000, whereas it should have read 0, 1, 10, 100, 1000, 10 0000, and 100 000. The correction has been made online as of Dec 14, 2015, and the print version is correct.

